# Analysis of adherence to HIV-positive quality of care indicators and their impact on service quality perceptions in patients: a Spanish cross-sectional study

**DOI:** 10.1186/s12955-020-01441-w

**Published:** 2020-06-15

**Authors:** A. Gimeno-García, A. Franco-Moreno, C. Montero-Hernández, S. Arponen, E. García-Carrasco, B. Alejos, D. Corps-Fernández, E. Gaspar-García, P. Galindo-Jara, M. García-Navarro, D. Varillas-Delgado

**Affiliations:** 1 Servicio de Medicina Interna, Hospital Universitario de Torrejón. Calle Mateo Inurria S/N. 28850, Torrejón de Ardoz, Madrid Spain; 2 Universidad Francisco de Vitoria, Carretera Pozuelo-Majadahonda km 1.800, 28223 Pozuelo de Alarcón, Madrid, Spain; 3 Servicio de Medicina Preventiva, Hospital Universitario de Torrejón, Calle Mateo Inurria S/N, 28850 Torrejón de Ardoz, Madrid Spain; 4 Centro Nacional de Epidemiología, Instituto de Salud Carlos III, Avenida Monforte de Lemos 5, 28029 Madrid, Spain; 5 Servicio de Medicina Interna, Hospital Perpetuo Socorro, Avda. Damián Téllez Lafuente, S/N, 06010 Badajoz, Spain; 6 Servicio de Cirugía General, Hospital Universitario de Torrejón, Calle Mateo Inurria S/N, 28850 Torrejón de Ardoz, Madrid Spain

**Keywords:** HIV, Acquired immunodeficiency syndrome, Quality indicators, Health care: patient satisfaction

## Abstract

**Background:**

Since the identification of human immunodeficiency virus (HIV) infection, there have been significant advances in its diagnosis and treatment, but there have been few contributions to the area of care quality. In 2010, the Spanish AIDS Study Group (GeSIDA) published the document “Health quality indicators of GeSIDA for the care of people infected with HIV/AIDS” in which standards are proposed for the purpose of improving and standardizing the assistance provided to people infected with HIV. The purpose of this study was to evaluate the degree of compliance with these indicators and to analyse whether adherence to the standards improves patient perception of care quality in terms of their satisfaction with the health care they have received.

**Methods:**

Compliance with GeSIDA indicators was analysed within a cohort of people living with HIV (PLHIV) in a hospital in the Madrid region. To evaluate patient perception, the External Consultation User Satisfaction Questionnaire (SUCE) was used, which is a tool that was previously validated in the Spanish population.

**Results:**

A total of 334 patients were included. The level of adherence to the indicators was 74.46%. The score on the SUCE questionnaire was 9.04 out of 10 (CI 95%: 8.90–9.19). Of the 47 indicators assessed, only 4 were related to satisfaction with health care.

**Conclusions:**

The levels of compliance with the indicators and patient satisfaction with health care were high. Adherence to quality indicators showed little relation to patient-reported satisfaction.

## Background

The desire to improve the quality of health care has led to the development of assessment tools [1]. Donabedian’s study [2] established the basis of the quality systems applied to health care and defined quality as “*an adaptation of the attention to the particular needs of each case*”, highlighting that the majority of health care evaluations are limited to a recounting of the actions undertaken, without taking into consideration their effects on health or the extent to which they meet the needs of patients. In recent years, demand has grown for health care that, in addition to being effective and evidence-based, is perceived as satisfactory and beneficial by the patient [3]. This perspective is especially important in patients with chronic diseases, such as infection with the human immunodeficiency virus (HIV), since clinical and physiological measurements provide valuable information for the physician but are often poorly correlated with functional capacity and patient welfare [4]. In the case of patients with HIV infection, the Spanish AIDS Study Group (GeSIDA) published a document in 2010 that includes 66 indicators of care quality. Currently, patients with HIV infection have a longer life expectancy than they did historically and accumulate more comorbidities, which means that health professionals need to address issues beyond virological suppression [5]. User satisfaction is one of the most relevant characteristics that can be used to measure the quality of a service and serves as the basis for the formulation of appropriate health policies [6, 7].

Information reported by patients allows the implementation of actions to improve health care [8]. Some very important elements of health care, such as the perception of the results from the patient’s perspective, are poorly represented in the current health care indicators [9]. In this context, it seems especially relevant to monitor whether there is a relationship between the quality of care achieved through compliance with the established quality standards and the quality of care as perceived by the patient.

The aim of this study was to evaluate the compliance with the quality indicators established by the GeSIDA in a cohort of people living with HIV (PLHIV) and to analyse the influence of those indicators on patient satisfaction. Although some articles have been published on the indicators of the quality of care in the HIV-positive population in different contexts and their relationships with different factors [10, 11], the impact of those indicators on patient satisfaction has not been analysed.

## Methods

### Study design

This was a cross-sectional observational study of an HIV-positive Spanish population.

### Patients

In the analysis of compliance with the quality indicators, all PLHIV over 18 years of age who started or continued follow-up during the study period between September 2011 and November 2017 were included.

For the analysis of patient-reported satisfaction, patients who were not participating in active follow-up at the time of the survey (due to death, transfer to another centre or loss to follow-up), those who were not able to respond (due to illiteracy, language barriers or poor baseline situation), and those who declined to participate were excluded.

The work was carried out in accordance with the Helsinki declaration of 1974 (updated in 2000) and current personal data protection regulations. It was approved by the Research Commission of the study centre and by the Ethics Committee of the hospital. All patients included in the satisfaction analysis signed the patient information sheet and provided informed consent.

### Quality indicators

The indicators of the quality of care for HIV-positive patients proposed by GeSIDA were used. There were 66 indicators published in 2010 [12], which were subsequently validated, demonstrating their reproducibility and feasibility [13]. The indicators that all HIV units should monitor to determine their status and identify necessary improvement measures are considered *relevant*. The indicators that must meet the established standard so that the unit can be accredited are called *basic* [12]. All the basic and relevant indicators were analysed except for three: indicators *9-Relevant contents of the initial assessment* and *33-Periodic consultation report* could not be evaluated because the necessary data were not available; neither indicator is considered relevant. Indicator *52-Specific treatment of chronic hepatitis C virus* (HCV) was not analysed because during the study period, the treatment of chronic hepatitis C was based on direct-acting antiviral drugs and not on interferon and ribavirin, as stated in the definition of the indicator.

The analysis of compliance with the indicators was carried out by the research team following the definitions established by the GeSIDA experts. The information necessary for the evaluation was obtained from the electronic medical records and was collected in a specifically designed database.

### Study variables

To achieve the main goal of the study, patient satisfaction was analysed by means of the survey “*External Consultation User Satisfaction Questionnaire”* (SUCE), a self-reported previously validated in the Spanish population [14]. It consists of 12 items with a response scale from 1, the worst rating, to 10, the best rating. It allows the independent evaluation of the clinical quality by analysing aspects related to care by health personnel and the administrative quality by analysing organizational and structural aspects. By means of ROC curves, 6.3 was established as a cut-off point to discriminate between satisfied and unsatisfied patients [15].

The questionnaires were offered in English and Spanish at the end of the consultation, and the patients answered them in a separate space, depositing them at the end in an container located in the administrative area of the centre.

Other relevant variables, such as age, sex, educational level, country of origin, date of diagnosis of HIV infection, transmission mechanism, CD4+ cell count and HIV viral load (VL), were analysed.

### Statistical analysis

All analyses were performed with the statistical program STATA 14 College Station, TX.

A descriptive analysis of the basic characteristics of all the patients was performed; frequency distributions were used for categorical variables, and means (standard deviations) or medians (interquartile ranges) were used for continuous variables, based on the normality of their distributions. These characteristics were compared between all patients and those participating in the survey with the chi-square test and Student’s t-test to assess the representativeness of the sample.

Forty-seven indicators of the quality of care were evaluated with their 95% parametric confidence intervals (CIs). An indicator was met when the 95% CI of the compliance percentage contained the value defined as the standard.

Linear regression models were used to estimate the differences in means and 95% CIs with regard to the effect of compliance with the indicators on patient satisfaction. For the regression analyses, the indicators calculated at the individual level were included for the patients who participated in the interviews.

To study which quality indicators were independently associated with satisfaction, multivariable regression models were constructed, in which independent variables were included in addition to the indicators age, sex, transmission mechanism, country of origin, CD4+ cell count and VL at the time the questionnaire was completed. Those variables with *p* <  0.05 were retained in the model.

## Results

A total of 334 patients with HIV infection were treated in the outpatient clinic between September 2011 and November 2017. Table 1 shows the descriptive analysis of the patients. A total of 64.1% of the patients were men. Forty-four were foreigners, and sub-Saharan Africa was the most frequent place of origin (24.4%). Fifty percent of the patients had been diagnosed before 2007 (IQR; 1996; 2013), and the most frequent infection risk category was heterosexual (43.2%), followed by parenteral drug addict (PDA) (28.4%) and men who have sex with men (MSM) (25.4%). On the first visit to the centre, the median age was 42 years (IQR: 35; 49), the median CD4+ lymphocyte count was 457 cells/mm^3^ (CI: 219; 685), and 40.4% had undetectable plasma VLs. The median follow-up time in the centre was 3 years (IQR: 1; 5).

**Table 1:** Characteristics of patients treated in the HIV program stratified by survey participation

	Lost to follow-up, transferred, dead	Did not respond to survey	Responded to survey	Total	*P* value
Number	93	77	164	334	
Sex					0.664
Male	61 (65.6%)	46 (59.7%)	107 (65.2%)	214 (64.1%)	
Female	32 (34.4%)	31 (40.3%)	57 (34.8%)	120 (35.9%)	
Transmission					0.005
PDA	35 (37.6%)	18 (23.4%)	42 (25.t6%)	95 (28.4%)	
Unknown	1 (1.1%)	5 (6.5%)	2 (1.2%)	8 (2.4%)	
MSM	15 (16.1%)	14 (18.2%)	56 (34.1%)	85 (25.4%)	
Heterosexual	42 (45.2%)	40 (51.9%)	62 (37.8%)	144 (43.2%)	
Blood transfusion	0 (0.0%)	0 (0.0%)	1 (0.6%)	1 (0.3%)	
Vertical	0 (0.0%)	0 (0.0%)	1 (0.6%)	1 (0.3%)	
Country of origin					0.024
Spain	56 (60.2%)	36 (46.8%)	107 (65.2%)	199 (59.6%)	
Other	37 (39.8%)	41 (53.2%)	57 (34.8%)	135 (40.4%)	
Age at first visit, years	44.2 (37.2;49.3)	41.6 (31.9;48.8)	42.1 (34.1;47.4)	42.3 (34.6;48.5)	0.645
Viral load at first visit					
Undetectable (< 50 copies/ml)	34 (36.6%)	39 (50.6%)	62 (37.8%)	135 (40.4%)	0.143
CD4+ cell count at first visit (cells/mm^3^)	413.0 (179.0;639.0)	491.0 (237.0;747.0)	474.0 (215.5;698.0)	457.0 (219.0;685.0)	0.227
Years of follow-up	1.0 (0.2;1.8)	4.3 (2.9;5.1)	3.6 (2.0;4.8)	2.9 (1.0;4.6)	< 0.001

*PDA* Parenteral drug addiction

*MSM* Men who have sex with men

**Fig. 1:**
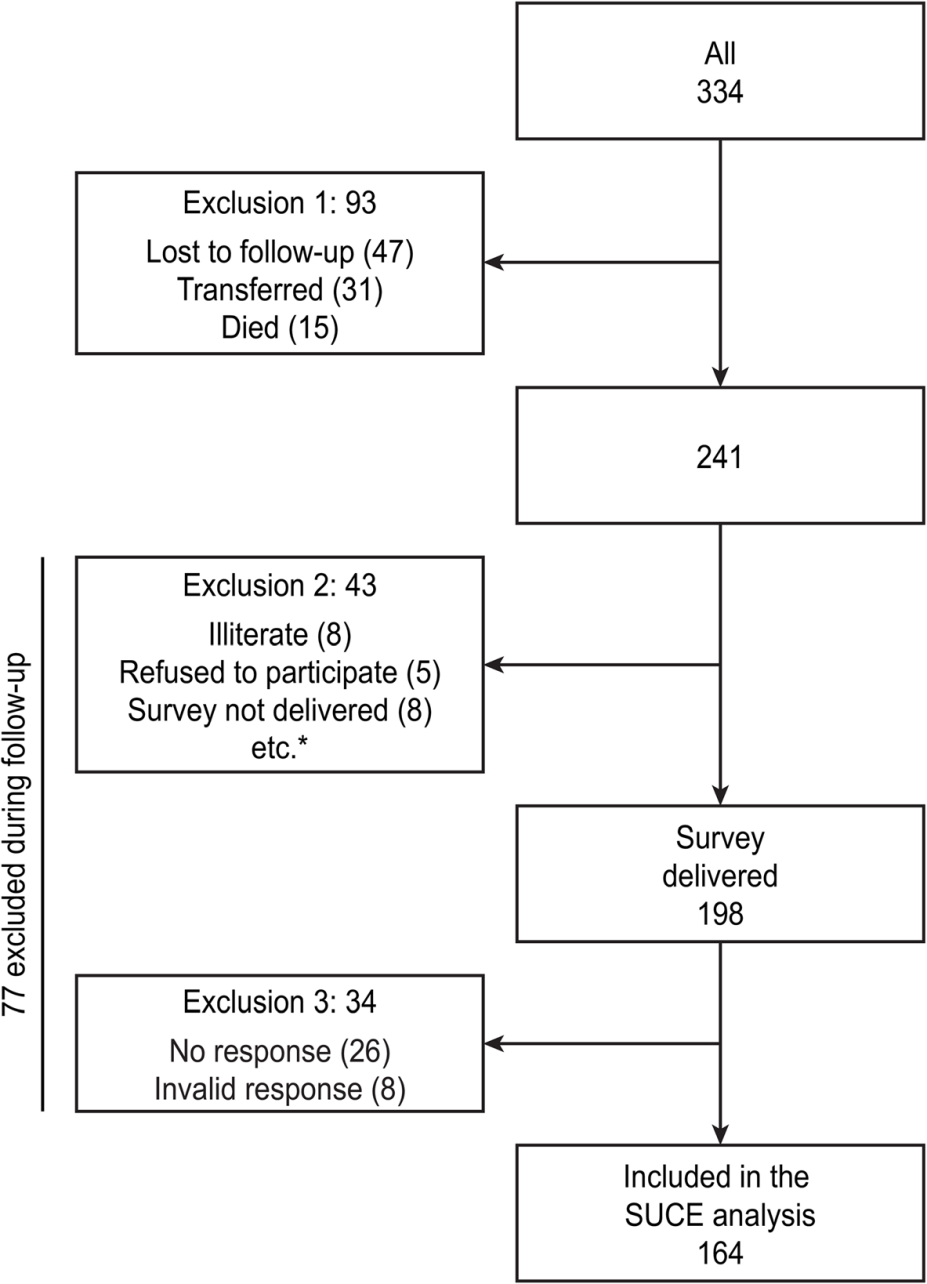
General outline of the study: patients included between 2011 and 2017

To calculate the quality indicators, 334 PLHIV treated in the outpatient clinic during the study period were included. At the time of the survey, 93 patients were not being followed up (lost to follow-up, transferred or died), and 43 patients were excluded due to illiteracy, language barriers, organic or psychiatric pathology that made it impossible for them to complete the questionnaire or refusal to participate. Of the 198 surveys administered, 172 were collected (87% response rate). In the satisfaction analysis, 164 patients were included because 8 surveys were not valid (Fig. 1).

There were differences between patients included and excluded in the survey analysis. The percentage of participation in the surveys was higher in patients with longer follow-up times (*p* <  0.001), MSM (*p* = 0.005) and patients of Spanish nationality (*p* = 0.038) (Table 1).

### Quality indicators and user satisfaction

**Fig. 2:**
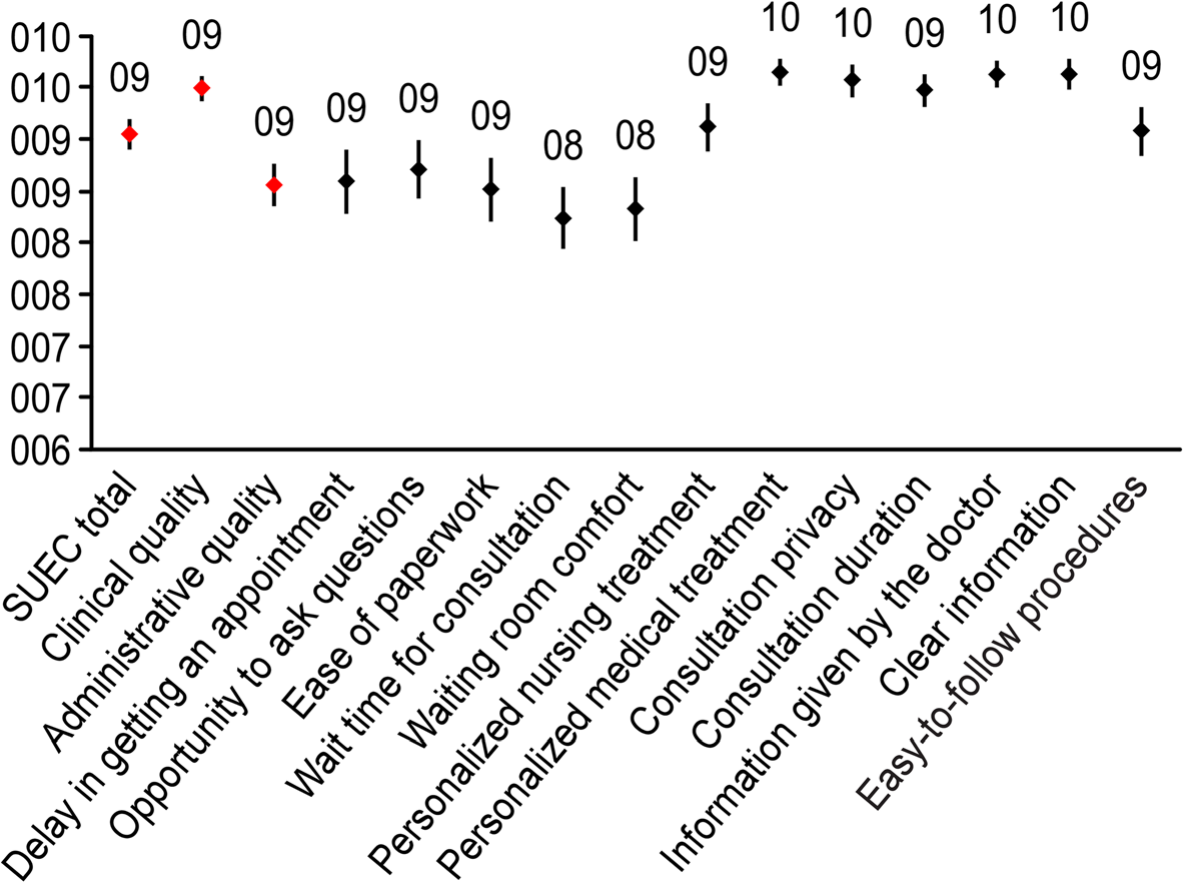
Results of the satisfaction questionnaire (SUCE) in the interviewed patients

Compliance with the quality indicators was high: of the 47 indicators evaluated, 35 met the established standard (74.46%). Table 2 shows the results of compliance with each of the indicators. The SUCE scale score was 9.04 (95% CI: 8.90; 9.19) (Fig. 2). Based on the proposed cut-off value of 6.3 [15], 98.16% of the patients were satisfied. The *Clinical Quality Factor Score* (9.48, 95% CI: 9.37;9 .60) was higher than the *Administrative Quality Score* (8.56, 95% CI: 8.35; 8.76). Table 3 shows the univariate analysis of quality indicators related to satisfaction (SUCE). Those related to greater satisfaction were *16-Periodicity of visits* [difference in means 95% CI: 0.62 (0.13;1.11)], *21-Vaccination against hepatitis A* [difference in means 95% CI: 1.14 (0.16;2.12)] and *23-Vaccination against pneumococcal infection* [mean difference 95% CI: 0.74 (0.10;1.38).

**Table 2:** Quality indicators for all patients evaluated

N	Indicator	Patients evaluated by the indicator	Patients who met the indicator				Standard set by GeSIDA	Compliance with the indicator in the study
		(*N*)	(*N*)	(%)	(CI 95%)		(%)	
1	Specialized doctor care			100			100%	Yes
2	Availability of diagnostic resources			100			Yes (all)	Yes
3	External pharmacy for dispensing drugs			No			Yes	No
4	Conditions of privacy and structural confidentiality			100			100%	Yes
6	Delay in referral to specialized care	47	42	89.4	76.9	96.5	100%	No
7	Late diagnosis of HIV in specialized care	106	28	26.4	18.3	35.9	< 25%	Yes
8	HIV diagnosis with previous negative serology	106	64	60.4	50.4	69.7	80%	No
10	Complementary tests in the initial assessment	334	322	96.4	93.8	98.1	95%	Yes
11	HIV plasma viral load	334	334	100	98.9	100	100%	Yes
12	Determination of lymphocyte subpopulations (CD4)	334	334	100	98.9	100	100%	Yes
13	Health education at initial assessment	332	187	56.3	50.8	61.7	95%	No
15	Indication of treatment with < 350 CD4 and without prior ART	117	3	2.6	0.5	7.3	< 10%	Yes
16	Periodicity of visits (regular follow-up)	218	193	88.5	83.5	92.4	85%	Yes
17	Basic renal study in HIV+ patients	212	209	98.6	95.9	99.7	100%	Yes
20	LTI detection	189	110	58.2	50.8	65.3	90%	No
21	Vaccination against hepatitis A	58	54	93.1	83.3	98.1	85%	Yes
22	Vaccination against hepatitis B	88	81	92.0	84.3	96.7	85%	Yes
23	Vaccination against pneumococcal infection	213	193	90.6	85.9	94.2	85%	Yes
24	Prophylaxis against *Pneumocystis jirovecii* and *Toxoplasma*	42	41	97.6	87.4	99.9	100%	Yes
25	Treatment and prevention of smoking	93	67	72.0	61.8	80.9	95%	No
26	Alcohol intake assessment	218	8	3.7	1.6	7.1	95%	No
29	Syphilis screening	149	68	45.6	37.5	54.0	70%	No
30	LTI treatment	25	23	92.0	74.0	99.0	95%	Yes
31	Loss to follow-up	255	14	5.5	3.0	9.0	≤ 5%	Yes
32	Recovery of failed appointments			84.9			85%	Yes
35	Adaptation of initial ART to the guidelines	119	119	100	96.9	100	95%	Yes
36	Initiation of ART in patients with symptomatic B/C events	33	32	97.0	84.2	99.9	90%	Yes
37	First visit after the establishment of ART	117	108	92.3	85.9	96.4	90%	Yes
38	Undetectable viral load (< 50 copies/ml) at week 48	106	102	96.2	90.6	99.0	80%	Yes
39	Treatment with Abacavir (ABC) without previous HLA-B 5701	77	0	0	0	4.7	0%	Yes
40	Treatment changes during the first year	98	20	20.4	12.9	29.7	< 30%	Yes
41	Record of adherence to treatment	312	266	85.3	80.8	89.0	95%	No
42	Study of resistance in case of virologic failure	45	41	91.1	78.8	97.5	90%	Yes
44	Average expenditure per patient in first treatment	13		8710.8*			**	Yes
45	ART in pregnant women with HIV	17	17	100	80.5	100	100%	Yes
47	Vertical transmission incidence	17	0	0	0	19.5	< 1%	Yes
49	Evaluation by CHILD or MELD for chronic liver disease	12	6	50.0	21.1	78.9	100%	No
50	Evaluation of hepatitis C virus coinfection	7	7	100	59.0	100	90%	Yes
54	HBsAg patients receiving effective treatment	12	12	100	73.5	100	90%	Yes
55	Ultrasound control in cirrhotic patients	8	4	50.0	15.7	84.3	90%	No
56	Cardiovascular risk assessment	212	120	56.6	49.6	63.4	90%	No
58	Patients with discharge report after hospitalization	80	80	100	95.5	100	100%	Yes
59	Reports of discharge of deceased patients in the hospital	12	12	100	73.5	100	100%	Yes
60	Follow-up in outpatient clinic after hospital discharge	74	74	100	95.1	100	100%	Yes
62	Overall mortality rate in patients in follow-up	334	15	11.7	8.7	24.9	≤ 25***	Yes
63	Mortality rate due to AIDS-related causes	334	3	3.2	1.0	9.8	Not established	
64	Continuing education			100			75%	Yes

* Cost in euros of initiating treatment in this population

** Median rates published in the corresponding year by GeSIDA (7506.5 (6556–9072)) [16]

*** Death rate per 1000 people/year

**Table 3:** Univariate analysis of quality indicators related to satisfaction (SUCE)

N	Healthcare quality indicator	SUCE
		Mean difference (CI 95%)
6	Delay in referral to specialized care	−0.26 (−1.33;0.81)
7	Late diagnosis of HIV in specialized care	−0.23 (− 0.81;0.35)
8	HIV diagnosis with previous negative serology	−0.10 (− 0.65;0.44)
10	Complementary tests in the initial assessment	0.01 (−1.07;1.10)
11	HIV plasma viral load	–
12	Determination of lymphocyte subpopulations (CD4)	–
13	Health education at initial assessment	0.24 (−0.06;0.54)
15	Indication of treatment with < 350 CD4 and without prior ART	–
16	Periodicity of visits (regular follow-up)	0.62 (0.13;1.11)*
17	Basic renal study in HIV+ patients	−0.57 (− 2.49;1.35)
20	LTI detection	0.09 (−0.27;0.44)
21	Vaccination against hepatitis A	1.14 (0.16;2.12)*
22	Vaccination against hepatitis B	0.49 (−0.41;1.40)
23	Vaccination against pneumococcal infection	0.74 (0.10;1.38)*
24	Prophylaxis against *Pneumocystis jirovecii* and *Toxoplasma*	−0.14 (−3.03;2.76)
25	Treatment and prevention of smoking	0.11 (−0.52;0.75)
26	Alcohol intake assessment	−1.20 (− 1.97; −0.44)*
29	Syphilis screening	0.03 (−0.35;0.41)
30	LTI treatment	−0.60 (−2.18;0.98)
35	Adaptation of initial ART guidelines to the guidelines	–
36	Initiation of ART in patients with symptomatic B/C events	–
37	First visit after the establishment of ART	−0.69 (−1.61;0.23)
38	Undetectable viral load (< 50 copies/ml) at week 48	−0.62 (−2.76;1.52)
39	Treatment with Abacavir (ABC) without previous HLA-B 5701	–
40	Treatment changes during the first year	−0.08 (− 0.87;0.71)
41	Record of adherence to treatment	0.33 (− 0.14;0.79)
42	Study of resistance in case of virologic failure	−0.48 (−1.85;0.90)
45	ART in pregnant women with HIV	–
47	Vertical transmission incidence	–
49	Evaluation by CHILD or MELD for chronic liver disease	1.67 (−1.34;4.67)
50	Evaluation of the hepatitis C virus coinfection	–
54	HBsAg patients receiving effective treatment	–
55	Ultrasound control in cirrhotic patients	1.25 (−3.23;5.73)
56	Cardiovascular risk assessment	−0.01 (− 0.34;0.31)

* *p*-value < 0.05

In contrast, those who met the *26-Evaluation of alcohol intake* had a lower level of satisfaction [95% difference in means 95% CI: − 1.20 (− 1.97; − 0.44)]. Foreign patients reported greater satisfaction than Spaniards [95% CI difference: 0.42 (0.12;0.71)] (Table 4).

**Table 4:** Univariate analysis of other factors related to satisfaction (SUCE)

Other factors	SUCE
	Mean difference (CI 95%)
Age	−0.01 (− 0.02;0.01)
Level of education	
None	0
Primary	0.26 (−0.24;0.75)
Secondary	0.02 (−0.47;0.50)
University	0.37 (−0.18;0.93)
Sex	
Male	0
Female	0.18 (−0.12;0.49)
HIV transmission category	
PDA	0
MSM	−0.31 (−1.02;0.39)
Heterosexual	−0.29 (− 0.97;0.40)
Country of origin	
Spain	0
Other	0.42 (0.12;0.71)*
Years since HIV diagnosis	−0.01 (−0.02;0.01)
CD4+ count (cells/mm^3^) at the time of submitting the survey	
< 200	0
200–500	0.23 (−0.36;0.82)
> 500	0.35 (−0.19;0.90)
Viral load (copies/ml) at the time of delivery of the survey	
< 50	0
50–100,000	0.07 (−0.36;0.51)
> 100,000	0.97 (−0.90;2.84)

* *p*-value < 0.05

Table 5 shows the multivariable analysis of satisfaction-related factors (SUCE). The final satisfaction model included the indicator 16-*Periodicity of visits* (regular follow-up) and country of origin.

**Table 5:** Multivariable analysis of satisfaction-related factors (SUCE)

	Mean difference (CI 95%)
	SUCE
Indicator 16	0.55 (0.08;1.02)
Country of origin (ref. Spain)	
Other	0.36 (0.06;0.66)

## Discussion

The baseline characteristics of the 334 patients treated at the centre were significantly different from those of other Spanish cohorts [17, 18], with a higher percentage of foreigners (40.42%), mainly from sub-Saharan Africa. This may account for the fact that the most common risk category was HTSX and not MSM, as in other published series. Although the male sex predominated, there were more women (36%) than usual in this type of study [18].

Of the 198 surveys administered, 172 were collected, with a response rate of 87%, which is much higher than that published in other works based on self-reported questionnaires [14, 19–21].

A low response rate may result in a response bias if the responding patients have significant differences from non-responders [22, 23]. Some authors have proposed that a response rate of at least 80% is acceptable [23, 24], while a response rate of 60% would not be adequate in terms of the representativeness of the sample [23].

Among the patients who participated in the survey, Spaniards accounted for a higher proportion, which is reasonable because the questionnaires were delivered in Spanish and English, and not all foreign patients speak those languages. The authors believe that by providing a separate space in which to complete the surveys and another in which to deliver them, both of which were outside of the consultation room, the participation of the patients was not biased.

### Quality indicators

Although the level of compliance with the quality indicators was high, there are areas for improvement. With regard to some indicators, for which the subjective feeling of compliance is high, the result for the indicator was lower than the recommended standard, probably because there is not always a written record in the clinical history of the actions performed, for example, indicators *8-Proof of previous HIV serology*, *13-Health education in the initial assessment*, *25-Treatment and prevention of smoking* and *41-Registration of adherence to treatment*.

There is another group of indicators related to the comorbidities of patients with which the level of compliance was suboptimal: *29-Syphilis screening*, *49-Evaluation by CHILD or MELD for chronic liver disease*, *55-Ultrasound control in cirrhotic patients* and *56-Assessment of cardiovascular risk*. Due to these results, the performance protocols have been modified, and reminders have been placed in the clinical history to improve compliance.

In the case of indicator *20-Detection of latent tuberculous infection* (LTI), which evaluates the performance of LTI screening in the initial assessment, the disagreement between the number of Mantoux tuberculin skin tests requested and performed stands out; the increasingly widespread use of interferon-γ-based techniques in our setting is significantly improving compliance, as it is a technique that is routinely performed with other analyses, which avoids the need for the patient to make two trips to the hospital.

The result of indicator *26-Evaluation of alcoholic intake* deserves special mention; the low level of compliance with this indicator is because in daily practice, questions about alcohol intake are asked of patients with a history of excessive consumption and not of all patients during regular follow-up as required in the definition of the indicator.

### User satisfaction

The SUCE score was 9.04 points, which is higher than that obtained with the same questionnaire in other Spanish hospitals [7, 25]. It is likely that the higher level of satisfaction is related to the fact that in the hospital conducting this study, each patient is always treated by the same physician. In satisfaction studies conducted in outpatient clinics of different specialties, patients have suggested that to improve satisfaction, they should be attended by the same doctor in successive visits [19, 20].

Based on the proposed cut-off value of 6.3 [15], 98.16% of patients were satisfied at the time of the interview. The low proportion of dissatisfied patients makes comparisons between satisfied and dissatisfied patients difficult. The *Clinical Quality Score* was higher than the *Administrative Quality Score*, which is a finding previously described by other authors who have worked with this questionnaire [7, 26].

### Quality indicators and user satisfaction

An association was found between the performance of check-ups and analyses at least every 6 months and patient satisfaction; this finding should be interpreted with caution because it is possible that patients who are more satisfied with health care more regularly keep their scheduled appointments and that compliance with this indicator is the result of greater satisfaction and not cause of it.

It was also found that patients who had been vaccinated against hepatitis A virus (HAV) and pneumococcus were more satisfied, but those who had received a recommendation for vaccination against hepatitis B virus (HBV) were not. This disagreement suggests that although statistically significant differences were detected, their clinical relevance is probably minimal.

Compliance with the alcohol intake assessment indicator was associated with less satisfaction because, as it was applied in this population, it identifies patients with extreme alcohol consumption habits. Excessive alcohol consumption is associated with worse patient-reported satisfaction [27, 28].

In this cohort, compliance with indicator *11-Determination of plasma VL at the first visit* was 100, and 88.14% of the patients had undetectable VLs when they responded to the survey, although having an undetectable HIV VL did not influence the level of satisfaction.

Some authors have analysed the complex relationship between quality management strategies and the satisfaction perceived by the patient and did not find any significant associations [6]. This fact can be explained by the low impact that the strategies implemented to increase the quality of care have on patient perceptions [6]. The direct personal relationship of the patient with the physician or the nursing staff is a powerful predictor of the patient’s perception [29, 30]. To comprehensively assist patients with HIV infection, care indicators should focus not only on scientific evidence and clinical practice guidelines but should also promote the best clinical practice in other areas, such as organizational aspects, doctor-patient relationships, patient safety and medical errors [9].

### Strengths and limitations

The quality indicators used in this study have been validated in the Spanish population, and have been shown to be reliable, with interobserver concordance levels greater than 95% [13].

Some studies evaluated compliance with quality standards and related their results to social, demographic, cultural and clinical factors, but no studies have contrasted these results with the level of satisfaction of the PLVIH as a user of the healthcare system. This perspective provides a more comprehensive evaluation of the quality of healthcare offered to patients.

The satisfaction questionnaire used was validated in the Spanish population and was found to have good psychometric properties. Its use enhances the reliability and validity of our results, unlike other results obtained with non-validated questionnaires [19, 20]. The high response rate also adds value, reducing the possibility of biases and increasing the representativeness of the sample. As it is a generic questionnaire for application in outpatient clinics, it was not possible to analyse aspects specific to HIV infection, such as the type of antiretroviral therapy (ART) used, ART dosage, etc.

Offering the questionnaires in English and Spanish may have led to participation bias because some patients were not able to read in either of those languages. Delivering them immediately after the physician’s evaluation allowed the patient to recall their most recent impression and improved the quality of the information collected. The timing of the survey avoided the recall bias that would be expected if the administration of the survey had been delayed and also prevented the patient’s perception from being influenced by experiences in other areas of the hospital. However, the possibility of a Hawthorne bias, which is the bias that occurs if healthcare staff members modify their habitual attitudes because they are aware that they will be evaluated, must not be overlooked; this limitation is inherent in this type of work. The small number of dissatisfied patients in our study limited the possibility of comparing satisfied and dissatisfied patients.

## Conclusions

This study demonstrates the need to analyse our clinical practice with regard to the care of HIV-positive patients to identify areas of improvement and increase the level of patient satisfaction with the care received.

HIV VL is used to measure the effectiveness of ART and is important for the patient’s health but is not related to their perceptions of their satisfaction with the health care they have received. The achievement of the health objectives proposed by scientific societies does not imply the fulfilment of patient expectations. High-quality health care requires that health care professionals satisfy both official requirements and patient needs.

## Data Availability

The datasets used and analysed during the current study are available from the corresponding author on reasonable request.

## Abbreviations

ART: Antiretroviral therapy
CI: Confidence interval
GeSIDA: Spanish AIDS Study Group
HAV: Hepatitis A virus
HBV: Hepatitis B virus
HCV: Hepatitis C virus
HIV: Human immunodeficiency virus
LTI: Latent tuberculous infection
MSM: Men who have sex with men
PDA: Parenteral drug addict
PLHIV: People living with HIV
SUCE: External Consultation User Satisfaction Questionnaire
VL: Viral load
